# Loss and revival of androgen receptor signaling in advanced prostate cancer

**DOI:** 10.1038/s41388-020-01598-0

**Published:** 2021-01-08

**Authors:** Nicolò Formaggio, Mark A. Rubin, Jean-Philippe Theurillat

**Affiliations:** 1 Institute of Oncology Research, Università della Svizzera italiana, Lugano, Switzerland; 2 Department for BioMedical Research and Bern Center of Precision Medicine, University of Bern and Inselspital, Bern, Switzerland

**Keywords:** Cancer, Cancer genetics

## Abstract

Targeting the androgen receptor (AR) signaling axis has been, over decades, the mainstay of prostate cancer therapy. More potent inhibitors of androgen synthesis and antiandrogens have emerged and have been successfully implemented in clinical practice. That said, the stronger inhibition of the AR signaling axis has led in recent years to an increase of prostate cancers that de-differentiate into AR-negative disease. Unfortunately, this process is intimately linked with a poor prognosis. Here, we review the molecular mechanisms that enable cancer cells to switch from an AR-positive to an AR-negative disease and efforts to prevent/revert this process and thereby maintain/restore AR-dependence.

## Introduction

**Fig. 1:**
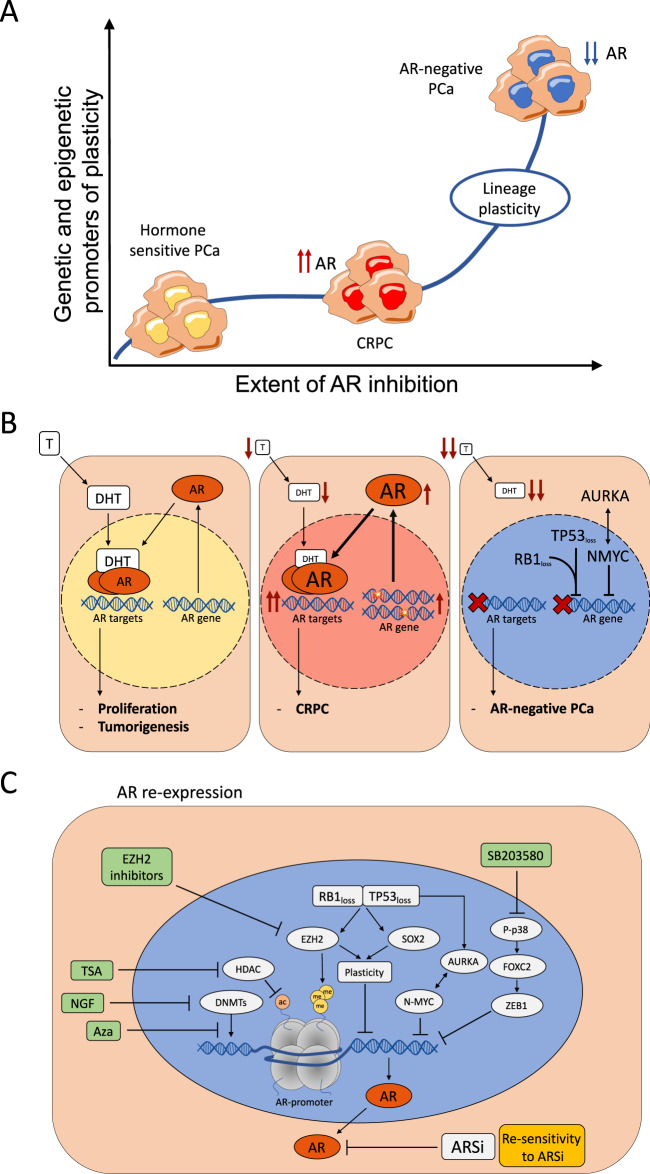
Control of AR expression during PCa progression. **a** The de-differentiation/lineage plasticity of androgen receptor (AR)-positive to AR-negative disease is likely dependent on the extent of AR inhibition (duration and/or type of inhibitors) and the existence of genetic and epigenetic adaption mechanisms (e.g., SOX2, TP53, RB1, N-MYC, EZH2), while resistance to androgen deprivation therapy (ADT) alone typically involves adaptation mechanisms in the AR pathway. **b** Prostate cancer growth and survival are dependent on testosterone (T). Testosterone converts locally to dihydrotestosterone (DHT), which binds and activates the AR and causes translocation to the nucleus. Here, AR promotes the transcription of cell cycle genes that promote cancer cell proliferation. ADT initially leads to the regression of the tumor but cancer cells often become resistant, referred to as castration-resistant prostate cancer (CRPC). At this stage, cancer cells adapt to the lower availability of DHT by acquiring gain of function mutations and amplifications on the AR gene. Upon exposure to ARSi (e.g., enzalutamide, abiraterone), prostate cancer cells may more likely undergo de-differentiation into AR-negative prostate cancer, which is associated with extensive rewiring of transcription and chromatin structure. **c** Scheme of different signaling pathways that can lead to AR epigenetic silencing and protein loss in prostate cancer cells. AR-independent prostate cancer cells can re-express AR protein through exogenous stimuli (in green) that block AR repression. Once re-activated, AR can re-sensitize those cells to ARSi. Aza azacitidine, NGF nerve growth factor, TSA Trichostatin A.

In prostate cancer, androgen steroid hormones bind to the androgen receptor (AR) and thereby trigger a key lineage-specific, oncogenic transcriptional program [1]. For many decades, this fact has been therapeutically exploited to treat de novo metastatic disease or recurrent metastatic disease after initial surgery or radiotherapy. Although androgen-deprivation therapies and/or the administration of first-generation competitive AR inhibitors prevent further tumor growth for a while, most patients develop resistance to the treatment and subsequently progress to castration-resistant prostate cancer (CRPC) (Fig. 1a) [2].

Over the last decade, more effective androgen receptor signaling inhibitors (ARSi, e.g., abiraterone, enzalutamide) have shown additional clinical benefit in patients with CRPC, indicating that CRPC remains largely dependent on the activation of AR signaling [3]. In line with this, CRPC cells adapt to androgens’ low availability by acquiring gain-of-function point mutations and gene amplification on the AR gene itself (Fig. 1b) [4]. Moreover, CRPC cells often acquire constitutively activated AR splice variants [5, 6]. Finally, upregulation of AR co-activators (e.g., NCOA2/3, TRIM24) [7, 8] and enzymes that promote intra-tumoral androgen production contribute to the re-activation of AR signaling in this setting [9, 10].

## Prostate cancer progresses to AR-negative fatal disease

The stronger inhibition of AR signaling through ARSi in recent years has led to an increase of metastatic prostate cancers that de-differentiate into AR-negative disease and consequentially no longer respond to the inhibition of the AR signaling axis (Fig. 1a). While being very rare in untreated patients, the incidence of AR-negative disease has increased in patients treated with ARSi [11, 12] and is expected to rise even more as ARSi are more widely used in the clinic in different disease stages. In Bluemn et al.’s work, the percentage of AR-negative tumors in patients with metastatic CRPC (mCRPC) increased from 11% (1998–2011) to 36% (2012–2016) after the introduction of potent ARSi such as enzalutamide and abiraterone [11]. Although there are no data yet, it is conceivable that even the use of potent chimeric small molecule AR degraders that will be entering clinical trials [13–15] could increase the percentage of AR-negative mCRPC.

There is little consensus about the treatment of AR-negative disease. The situation is further complicated because many patients with therapy-induced AR-negative disease are not diagnosed as the confirmation requires the assessment of current tumor tissue. That said, the treatment of confirmed AR-negative disease with neuroendocrine differentiation, the most common subtype of AR-negative mCRPC, is a platinum-based regimen similar to those employed for the treatment of other neuroendocrine small cell carcinomas. Unfortunately, response rates to cisplatin/carboplatin combinations with either docetaxel or etoposide are relatively high, but not durable [16–18]. Consequently, the prognosis is very poor with a mean survival that can vary from 12 months [19] to 36 months [17, 20], depending on the report. Thus, understanding the occurrence of AR-negative prostate cancer has become an important and urgent clinical need in the field. Here, we review the molecular mechanisms that enable cancer cells to switch from an AR-positive to AR-negative disease and efforts to prevent or revert this process and thereby sustain or restore AR dependence.

## Manifestations of AR-negative disease

The loss of AR expression during prostate cancer progression occurs as part of a larger cellular rewiring process that is paralleled by dramatic changes in cellular differentiation [21]. As AR-negative prostate cancer incidence increases, different subtypes have emerged that exhibit various cellular features. The distinction among different differentiation states based on morphological and molecular peculiarities is not in every case clear, and in some instances, different features and gradual changes co-exist within a single tumor nodule [22]. The classification of AR-negative prostate cancers remains thus a major challenge in the field.

Among the heterogeneous group of AR-negative tumors, the historically best-known subtype is neuroendocrine prostate cancer (NEPC), also referred to as small cell PCa [22, 23]. This subtype expresses abundant neuroendocrine and basal-like proteins, while the expression of AR-regulated luminal and epithelial markers is being lost [22]. Histologically, this subtype ranges from well-differentiated neuroendocrine tumors to de-differentiated cancers, frequently displaying small cell morphology [24]. Interestingly, other and rare histological subtypes that include squamous differentiation have been described as well [25].

More recent functional studies have demonstrated that NEPC typically arises from AR-positive conventional adenocarcinoma through a transdifferentiation process. Most notably, Zou et al. showed in lineage tracing mouse model studies that the neuroendocrine features arise from transdifferentiation of luminal cells [26]. Genetic inactivation of p53 increased neuroendocrine markers expression (e.g., synaptophysin) and decreased the response to abiraterone. Using a YFP tracer under an Nkx3.1 luminal specific promoter, the study revealed that nearly all the tumors with neuroendocrine markers also expressed YFP, proving evidence of their luminal-epithelial origin. Besides, androgen-sensitive prostate adenocarcinoma cells have been shown to exhibit neuroendocrine differentiation in androgen-depleted cell medium, thereby implying that castration actively promotes the development of NEPC [27]. The neuroendocrine phenotype emerges at least in part through suppression of AR signaling as the latter increases the expression of the neuronal transcription factor BRN2 [28]. Accordingly, BRN2 is inversely correlated with AR activity, as AR can directly suppress BRN2 expression [28]. Moreover, BRN2 can modulate SOX2 activity, a key driver of cellular plasticity discussed below [28]. Orthogonal evidence for transdifferentiation comes from recent cancer genomics studies. NEPC harbors in many instances genetic alterations that are reminiscent of AR-dependent CRPC. Among these, highly recurrent AR mutations and *TMPRSS2-ERG* gene fusions [20, 29–31].

Interestingly, there is an emerging link between the activation of Wnt/β-Catenin signaling and AR-negative disease as the Wnt/β-Catenin target genes FOXA2 and MYCN have been shown to promote neuroendocrine transdifferentiation [32–36]. Moreover, in vitro studies suggest that active β-Catenin in PCa cells increases neuroendocrine-specific protein, such as NSE and chromogranin A [37]. In line with this, inhibition of Wnt/β-Catenin signaling has been shown to reduce neuroendocrine transdifferentiation in vitro [37]. Finally, Wnt-11 has been observed to be upregulated in NEPC and functionally linked to transdifferentiation in vitro [38].

Besides NEPC, other subtypes of AR-negative disease have emerged. A double-negative subtype (negative for both AR and neuroendocrine markers) that is more intimately linked to the use of new-generation inhibitors of the AR-pathway has been described more recently [11]. The percentage of AR double-negative tumors has risen from 5% to more than 20% in the last 10 years, making it the most frequent subtype of AR negative prostate cancers [11]. This subtype’s proliferation is supported by increased autocrine FGF signaling that promotes at least part of the proliferation through activation of MAPK pathway [11]. In line with this, double-negative cancer cells are sensitive to FGF and MAPK signaling pharmacological inhibition.

Recently, a new subtype of mCRPC has been identified by chromodomain helicase DNA-binding protein 1 (CHD1) loss. Cancer cells with CHD1 deficiency have strong chromatin landscape alteration, which allows the outset of ARSi-resistant clones if challenged with enzalutamide [39]. This subtype of tumors often has increased glucocorticoid receptor (GR) levels and sustained GR signaling. Importantly, GR inhibition in the CHD1-deficient setting can restore enzalutamide sensitivity [39], suggesting that GR upregulation is critically involved in resistance to ARSi.

Recently, Labrecque et al. distinguished five different subtypes of mCRPC based on RNA expression of AR and the most common neuroendocrine markers: AR-high tumors (ARPC), AR-low tumors (ARLPC), amphicrine tumors with both the expression of AR and NE markers (AMPC), double-negative tumors (DNPC), and tumors with small cell and NE features without AR expression (SCNPC) [40]. Despite recent progress, functional commonalities and differences among AR-negative subtypes concerning clinical features and specific therapeutic opportunities remain to be investigated in the upcoming years. Future improvements will definitely require coordinated teams with multidisciplinary expertise, molecularly based biomarker inclusion, and an accurate selection of the patients, as described in a recent NCI Sponsored Workshop [41].

As mentioned above, the different subtypes are not always clearly separated from each other and likely also not stable over time [40]. Because of this, it has been assumed that they rather represent different stages of a continuous path toward de-differentiation featuring acquisition of stem cell features, AR-loss and -independence at its very end [40, 42, 43]. Thus, the current review focuses on the more general molecular mechanisms involved in the path to AR-negative disease. Because AR-negative mCRPC with neuroendocrine features is the most well-studied subtype, we will often refer to it in the text as an example of AR-negative disease without implying that the data cited are exclusively valid for NEPC.

## Genetic mechanisms facilitating AR downregulation and lineage switching

The de-differentiation from AR-positive to AR-negative disease involves cellular plasticity and extensive rewiring of transcription [41, 44–46]. The process likely requires the involvement of multiple genetic driver alterations intimately linked with prostate cancer progression, effective inhibition of the AR signaling axis, and time to enable the downstream epigenetic effectors to continuously reprogram the transcriptome of cancer cells depending on conditions [47] (Fig. 1). Factors and mechanisms described below enable cancer cells to be in a plastic state. This plasticity makes them disposed to react according to external stimuli continuously.

Multiple oncogenes and tumor suppressor genes have been linked with lineage switching from AR-positive to AR-negative prostate cancer (Fig. 1). Importantly, different studies have demonstrated that genetic alterations have to act in concert to enable lineage switching. Most notably, only the combined loss of the tumor suppressors RB1 and TP53 in genetic mouse models enables the lineage switching from AR-dependent luminal tumors to AR-independent neuroendocrine and basal-like cancers [44, 45]. In line with this, the transformation of human basal prostate epithelial cells to small cell NEPC in immunodeficient mice depends on the joint loss of RB1 and TP53 function [48]. In human tumor tissues, combined genetic alterations in both RB1 and TP53 have been observed more frequently in NEPC compared to CRPC (53% vs 13%) [23, 31]. At the molecular level, RB1 and TP53 co-silencing can upregulate both the histone methyltransferase EZH2 and the transcriptional pioneering factor SOX2. Acting together, they change the transcriptional output of prostate cancer cells and lead to the loss of luminal and epithelial markers, such as AR, and the increase in the expression of basal and neuroendocrine-related genes [44, 45,49–51] (Fig. 1b, c).

**Fig. 2:**
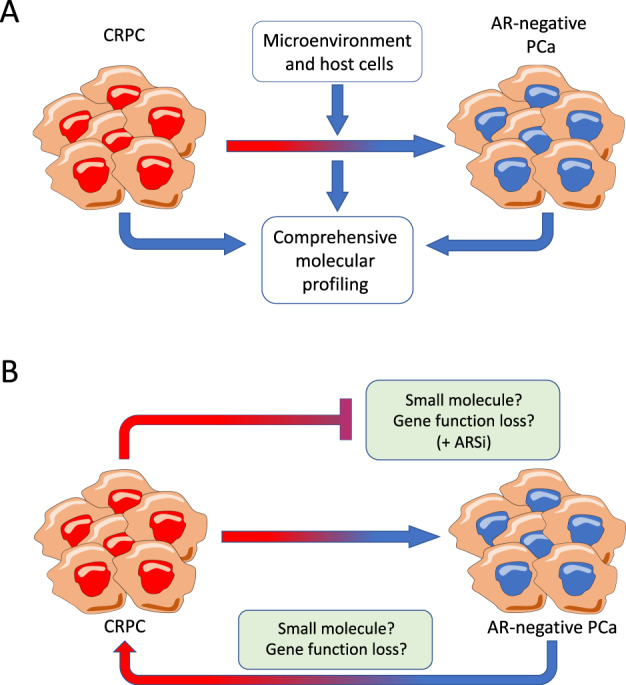
Perspective functional approaches related to AR expression in advanced PCa. **a** Characterization of cellular, microenvironmental, and molecular changes associated with the transition of AR-positive to AR-negative disease. **b** Identification of susceptibilities and small molecule compounds that target AR-negative disease, the switch of AR-positive to negative disease, and the reversal of AR-negative to -positive disease.

Notably, there is not a complete concordance between RB1 and TP53 combined loss and AR-negative diseases [52]. In fact, 40% of tumors that harbor both these two genetic alterations are classified as AR-active adenocarcinomas. All these findings suggest that the combined loss-of-function of TP53 and RB1 per se is neither essential nor sufficient to promote in every case the switch to AR-negative disease [52]. The determination of additional factors contributing to lineage plasticity and de-differentiation remains largely unexplored territory (Fig. 2). Another factor frequently neglected in the interpretation of molecular findings is that these represent only a point in time and plasticity drivers (e.g., TP53/RB1) may require a considerable period to complete epigenetic reprogramming. Thus, it remains largely unknown if AR-positive, TP53/RB1 mutated tumors may shutdown AR signaling during later time points.

As mentioned above, also the activation of oncogenes can induce the de-differentiation to AR-negative prostate cancer. The oncogene NMYC is a key regulator of prostate tumorigenesis involved in de-differentiation to AR-negative prostate cancer [34, 53]. Indeed, NYMC overexpression can increase neuroendocrine markers and histone methyltransferase EZH2 expression levels, which in turn can downregulate AR expression through repressive H3K27me3 histone marks [35]. Berger et al. functionally characterized in more detail the gene expression and chromatin changes in LNCaP cancer cells upon overexpression of NMYC [34]. The results reveal that NMYC co-opts members of the AR transcriptional network (e.g., FOXA1, HOXB13) on the path toward induction of neural differentiation and AR independence [34]. Together, these results strongly confirm the role of NMYC as a key master regulator toward AR-negative prostate cancer.

There is an intimate interplay between NMYC and AURKA, a critical kinase and oncogene involved in cellular division. Different groups have shown multiple positive feedback loops linking AURKA and NMYC and sustaining one another’s expression [30, 54]. In almost all the cases where AURKA was overexpressed (>90%), the authors also found NMYC overexpression, proving their close connection and their relevance for AR-negative PCa [30] (Fig. 2). If overexpressed in prostate cancer cells, both of them can increase the expression of neuroendocrine markers and downregulate luminal markers, including AR [30]. Mechanistically, AURKA decreases AR levels by activating CHIP, an E3 ubiquitin-ligase that degrades AR through the proteasome [55].

The oncogenes and the tumor suppressors cited above may be part of a larger, highly interconnected network. For example, AURKA can inactivate p53 by phosphorylation and p53 activation can decrease AURKA transcription [56]. Moreover, TP53 loss activates CDK2, which inhibits RB1. RB1 loss or inhibition releases E2F, directly increasing AURKA, NMYC, and EZH2 expression [56]. Given the functional overlap between these tumor suppressors and oncogenes, it is conceivable that various combinations in concert may induce lineage plasticity from AR-positive to AR-negative disease.

## Transcription factors involved in reprogramming

Multiple transcription factors involved in cellular plasticity and AR independence have been linked to prostate cancer progression to AR-negative disease (Fig. 1c). There is a striking overlap between these transcription factors’ involvement and the Yamanaka transcription factors that in concert induce pluripotency in normal human cells. SOX2 is a transcription factor involved in maintaining self-renewal features and induces cellular reprogramming through its ability as a pioneer transcription factor to open the chromatin and facilitate gene expression [57]. Its expression increases in NEPC compared to CRPC, especially with TP53 and RB loss [45] (Fig. 1). Moreover, lineage plasticity induced by combined loss of RB1 and TP53 is in part due to SOX2 activity. Accordingly, the de-differentiation process that leads to androgen deprivation resistance can be blocked by restoring p53 or RB1 activity as well as by SOX2 silencing [45]. Besides, SOX2 has also been described as the main BRN2-target gene for neuroendocrine differentiation in Bishop et al.’s work [28].

Another Yamanaka’s factor, OCT4, is implicated in the de-differentiation process to AR-negative prostate cancer. OCT4 levels increase during neuroendocrine differentiation, drive AR-independence, and consequentially decreases the sensitivity toward pharmacological AR blockage [58–60]. Moreover, in Mu et al.’s work, OCT4 expression increases in cells with RB1 and TP53 loss [45]. In contrast to SOX2, OCT4 overexpression seems not sufficient to promote resistance to enzalutamide.

Possibly, there is also a role for MYC in prostate cancer progression. Its specific genetic locus is predominantly amplified within the 8q-arm in CRPC [61] and functional studies also suggest involvement from castration-sensitive to -resistant disease [62]. Notably, MYC and active, myristoylated AKT have been shown to transform normal human prostate epithelial cells into poorly differentiated prostate adenocarcinomas in immunodeficient mice [63]. In this model, further differentiation to NEPC is critically dependent on p53 and RB1 loss [48]. Recently, Kwon et al. showed that MYC’s overexpression, together with a constitutively activated AKT (caAKT), can drive lineage plasticity in human luminal cells–derived organoid [64]. As a plasticity consequence, MYC- and caAKT-overexpressed organoid showed heterogeneous expression of AR and neuroendocrine markers [64]. Nevertheless, it remains to be determined if the de-differentiation can also occur in the absence of MYC.

NANOG is another pluripotency-inducing transcription factor that has been linked to therapy resistance and tumor progression. In prostate cancer, NANOG overexpression leads to enhanced clonal growth, tumor regenerative capacity, and resistance to castration [65, 66]. Its presence seems to disrupt AR and FOXA1 signaling [66]. That said, MYC’s and NANOG’s involvement in the terminal de-differentiation to AR-negative prostate cancer remains to be elucidated. In contrast, the Yamanaka factor KLF4 has a tumor-suppressive role in prostate cancer. Its expression in prostate stem cells blocks malignant transformation, while its loss is associated with disease progression [67].

Finally, Onecut2 is another transcription factor involved in cellular differentiation that has been recently associated with lethal prostate cancer and the downregulation of the AR transcriptional program. Onecut2 expression induces neuroendocrine markers, while its inhibition or silencing decreases tumor growth and metastasis formation in mice [68]. In the same paper, the authors showed that Onecut2 is directly repressed by REST, a transcription factor known to be lost during neuroendocrine differentiation [69]. Moreover, it is also a direct activator of PEG10, which is identified as a master NEPC regulator [70]. Taken together, these findings show the multitude of connections between transcriptional reprogramming toward a more pluripotent state and the de-differentiation process in prostate cancer.

## Epigenetic factors in reprogramming and AR silencing

The different driver genes and transcription factors outlined above cooperate to de-differentiate prostate cancer cells to an AR-negative state (Fig. 1). Over time, this cooperation induces common downstream epigenetic factors that can reprogram prostate cells toward an AR-negative state that involves extensive rewiring of many cellular pathways (cell–cell adhesion, development, EMT, and stem cell programs) [31, 35, 44, 58]. Accordingly, many different genetic alterations (e.g., TP53, RB1, NMYC, AURKA) may induce similar chromatin and transcriptional changes, ultimately explaining why it is easier to identify AR-negative disease based on transcriptional or epigenetic changes rather than genetic modification [31]. Along those lines, the evaluation of genome-wide CpG-rich methylation allows much better segregation of patients with CRPC and NEPC than specific genomic alterations [31]. Moreover, loss of TP53 and RB1 in human prostate epithelial cells can deeply alter their chromatin structure. Notably, hyper-accessible regions in TP53/RB1 deficient cells were enriched for genes related to neuroendocrine differentiation, while hypo-accessible regions were enriched for luminal and epithelial markers [48].

One of the most important upregulated epigenetic modifiers during disease progression is EZH2, a histone methyltransferase whose mRNA and protein levels are strongly associated with prostate cancer progression [71]. Moreover, EZH2 levels dramatically increase from CRPC to NEPC disease [31, 72]. EZH2 affects the transcriptional rewiring process by generating H3K27me3 repressive marks on specific gene promoters, including AR itself [31] (Fig. 2). AR-negative cell lines, such as DU145 and PC3, have also increased levels of repressive H3K9me2 marks and a reduction of active marks (H3K4me3 and H3K9ac) at the AR gene promoter [73]. Besides, AR transcription is further downregulated through methylated CpG islands at the AR promoter [74]. Despite being very clear in AR-negative cell lines, the epigenetic silencing of AR in advanced human CRPC is not deeply understood.

Finally, other key chromatin regulators are the SWI/SNF complex members, whose functions are strictly associated with euchromatin [75]. Changes in the SWI/SNF complex members occur especially during embryonic development and neuronal differentiation [76, 77]. NEPC recently showed the presence of neural-specific subunits of SWI/SNF complex (BAF53B and BAF45B), which were absent in benign prostate, localized prostate cancer, and CRPC. Alterations in its subunits composition change interaction partners of this complex, modifying chromatin accessibility between NEPC and CRPC [78]. Importantly, it remains to be determined if these changes in SWI/SNF complex composition are a consequence of the neuroendocrine differentiation or have a key function in driving de-differentiation to AR-negative prostate cancer. Together, these findings suggest a strong role of epigenetic modifications in the lineage plasticity and loss of AR expression in disease progression.

## Re-activation of AR expression in AR-negative prostate cancer

Because the AR pathway represents such a key therapeutic contact point in prostate cancer, researchers have set out to search for means to restore AR signaling in AR-negative prostate cancer (Fig. 2). As mentioned above, induced lineage plasticity mediates the onset of AR-negative prostate cancer. For this reason, silencing or inhibiting transcription factors of cellular plasticity, such as SOX2, could have an impact on AR re-expression. In line with this, SOX2 silencing abrogates the capability of cells with TP53 and RB1 loss to develop lineage plasticity and to express neuroendocrine markers [45].

Alternatively, drugs that affect epigenetic silencing in different ways have been successfully used to re-express AR in AR-negative cell lines. Among these are drugs that block DNA methylation (azacytidine) and increase active (HDAC inhibitors Trichostatin A) or diminish repressive histone marks (EZH2 inhibitors) at the AR promoter [73, 74, 79] (Fig. 2). Most notably, various EZH2 inhibitors (DZNep, GSK126, EPZ6438) have been reported to upregulate AR protein expression in human NEPC cell lines NCI-H660/MDA PCa 144-13 and in mouse prostate epithelial cells with ablation of Rb1 and Trp53 [44, 73]. That said, the pharmacological inhibition of EZH2 does not seem sufficient to re-gain AR positivity in recently generated patient-derived NEPC organoid lines [80].

Moreover, indirect perturbations have shown the ability to restore AR expression as well. Among these are the administering of the nerve growth factor (NGF) to DU145 cells [81] (Fig. 2). NGF has two receptors, p75^NTR^ and TrkA. p75^NTR^ is known in prostate cancer field since its tumor suppressor features [82]. Moreover, its expression decreases during disease progression. Conversely, Trka activation has shown oncogenic features in prostate cancer [83]. Although the authors did not explain the mechanism of NGF-mediated AR re-expression in prostate cancer cells, NGF administration can downregulate DNA methyltransferases in other cells [84]. It is tempting to speculate that NGF treatment could restore AR through downmodulation of DNA methylation.

More recently, the Forkhead Box C2 protein (FOXC2) has been shown to modulate AR in both AR-positive and -negative cell lines. Generally, its expression is negatively correlated with AR presence. Indeed, its silencing in AR-negative DU145 cells can strongly re-establish AR protein expression. The effects on FOXC2 targets are mediated by Zeb1, a known transcriptional repressor. Since FOXC2 is directly activated by P-p38 phosphorylation, inhibition of this pathway by the p38 inhibitor SB203580 can exert the same effect in restoring AR expression [85] (Fig. 2). Interestingly, SB203580 administration can also decrease SOX2 levels in AR-negative cells, possibly linking p38 activity, SOX2 expression, and cellular plasticity [85].

At this point, the studies mentioned above provide only incomplete insights into the feasibility and relevance of AR re-expression in a few cell line models. Moreover, the clinical relevance is yet to be fully discovered. Nevertheless, these findings confirm that it is possible to revert at least in part the process that leads to de-differentiation and AR negativity.

## Consequences of AR re-expression

The question remains as to what the functional consequences are of AR re-expression (Fig. 2). The AR translocation upon ligand binding from the cytoplasm to the nucleus seems still functional in DU145 cells upon NGF administration [81]. Moreover, in the same cell line, AR resumed by FOXC2 silencing can induce a massive reduction in self-renewal potential and stem cell proprieties, features previously linked to AR independence and prostate cancer progression [85].

The AR gene has been exogenously transduced into AR-negative prostate cancer cell lines to study the consequences of AR expression in this setting. Although somewhat artificial, it could be useful to understand better which functionality AR can have in an AR-negative cell context. For example, in DU145 cells exogenously expressing AR leads to the upregulation of PSA [86] and severely damps cell migration and invasion through expression of genes related to cell–cell and extracellular matrix interaction (e.g., integrins [87] and chemokine receptor [88]), potentially explaining the slowdown of cell migration and invasion. Similarly, PC3 overexpressing AR under a natural AR promoter suppresses tumor growth, metastasis formation, and cell invasion both in vitro and in vivo [89]. The paradoxical tumor-suppressive effects induced by the relatively high AR expression in PC3 and DU145 cells are reminiscent of the antitumor responses triggered by supraphysiologic androgen levels in recent clinical studies [90–93].

Although few reports describe the consequences of AR revival in prostate cancer cells, results seem to be concordant. Conversely, AR re-expression may decrease the levels of transcription factors that were initially involved in the shutdown of its expression. For example, it has been shown that AR can directly bind the enhancer element within the SOX2 promoter in prostate cancer cells, resulting in the inhibition of SOX2 transcription [94]. Taken together, these data suggest that AR re-expression in AR-negative cell lines induces tumor-suppressive properties by negatively modulating cellular proliferation, migration, and invasion in a DHT-dependent manner [95]. That said, it remains largely unclear how generalizable these findings are and if AR re-activation will suppress the oncogenic potential of AR-negative prostate cancer or in some cases rather boost tumor growth.

## Re-sensitization to antiandrogens

Prostate cancer is almost uniformly dependent on androgens at its initial presentation [96], while the loss of AR expression during disease progression inevitably results in resistance to standard treatments that inhibit AR signaling. Conceivably, the restoration of AR expression in the latter setting may also re-establish the sensitivity toward inhibition of AR signaling (Fig. 2). As discussed previously, RB1 and TP53 loss can generate plasticity, especially upregulating SOX2 and EZH2, leading to androgen-independent tumor growth [44, 45]. Indeed, EZH2 inhibition or SOX2 silencing in AR-independent cell lines can re-establish sensitivity to the antiandrogen enzalutamide in vitro and in vivo [44, 45]. Similarly, the re-expression of AR by inhibition of the p38 signaling axis can also sensitize DU145 cells to enzalutamide in vivo [85]. Also, azacytidine can restore AR expression in AR-negative PC3 cells and azacytidine treatment followed by the antiandrogen bicalutamide blunts in vivo tumor growth [97]. Together, these data indicate that the re-expression of AR in AR-negative cells could revitalize the activity of AR inhibitors.

## Perspective

The development and clinical success of more efficient AR signaling inhibitors come with the drawback of an increased prevalence of highly aggressive, AR-negative prostate cancer. Lineage plasticity as a resistance mechanism will likely further increase in the future with the clinical implementation of potent chimeric small molecule AR degraders [13]. Between them, the oral available AR PROTAC degrader ARV-110 is just entered in phase 1 clinical trial for mCRPC [15] (NCT03888612). That said, this disease entity needs to be more extensively studied and functionally characterized in different model systems. Over the last few years, an increasing number of additional patient-derived organoid and xenograft models have been developed [80, 98, 99]. These model systems may help to further characterize signaling pathways and chromatin structure of AR-negative disease. Importantly, new model systems that easily switch from AR-positive to AR-negative could be developed and subsequently used to identify additional key molecular features associated with the switch from AR-positive to -negative prostate cancer (Fig. 2a). These could be further validated in in vivo models, such as the LTL331 model, that transdifferentiate from adenocarcinoma to NEPC after castration [100].

Mouse models may help to elucidate factors in the tumor microenvironment or specific cell types of the host that contribute or prevent the switch from AR-positive to -negative disease (Fig. 2a). In line with this, it has been already demonstrated that cancer-associated fibroblasts and prostaglandin-related inflammatory responses can contribute to NEPC progression [101, 102]. While genetically engineered mice with ablation of Tpr53, Rb1, and Pten may be used to study host-related factors, the syngeneic TRAMP-C model may represent a less cost and labor-intensive alternative in this regard [103].

The models mentioned above may also help develop new therapeutic approaches to treat or prevent the occurrence of AR-negative prostate cancer (Fig. 2b). Indeed, AR-negative prostate cancer cells have been shown to respond to AURKA [30], FGFR, or MAPK inhibitors [11], in line with the notion that these pathways are activated as a result of the lineage switch. More comprehensive small molecule and loss-of-function screens may uncover additional therapeutic contact points in AR-negative prostate cancer.

Over the last few decades, many researchers have pursued endeavors to re-activate AR expression in progressed prostate cancer cells (Fig. 2b). Most studies provided at this point are rather incomplete and give preliminary insights into this concept without solid in vivo validation experiments. Future efforts may take advantage of much greater availability of clinically relevant models derived from patients or genetically engineered mouse models that capture better the variations of AR-negative disease and the feasibility of restoring AR expression and the subsequent susceptibility to AR pathway inhibitors.

In a treatment naïve setting, the upfront inhibition of key factors involved in the lineage plasticity may render responses to ARSi more durable (Fig. 2b). Such a combinatorial approach may be used for patients at higher risk of developing AR-negative disease (e.g., presence of *TP53* and *RB1* mutations). The development of cell line models that switch easily from AR-positive to -negative disease may be useful to search for such combinatorial approaches. Complementary, loss-of-function screens may nominate effective therapeutic targets as well. Possibly, these efforts may identify target proteins that function in both oncogenic AR signaling and the induction of cellular plasticity to AR-negative disease. For example, the AR activator TRIM24 has been recently also involved in the activation of SOX2 [104, 105].

In conclusion, AR-negative prostate cancer incidence will further rise as our ability increases to abrogate AR signaling. The characterization of this currently fatal disease will hopefully contribute to the development of better treatment options for patients facing AR-negative disease and combination therapies to prevent disease progression under ARSi treatment.
